# Supplementary figures and images for: Spinal cord injury regulates circular RNA expression in axons
Source: Front Mol Neurosci. 2023 Aug 24;16:1183315. doi: 10.3389/fnmol.2023.1183315 (PMC10483835; doi:10.3389/fnmol.2023.1183315)

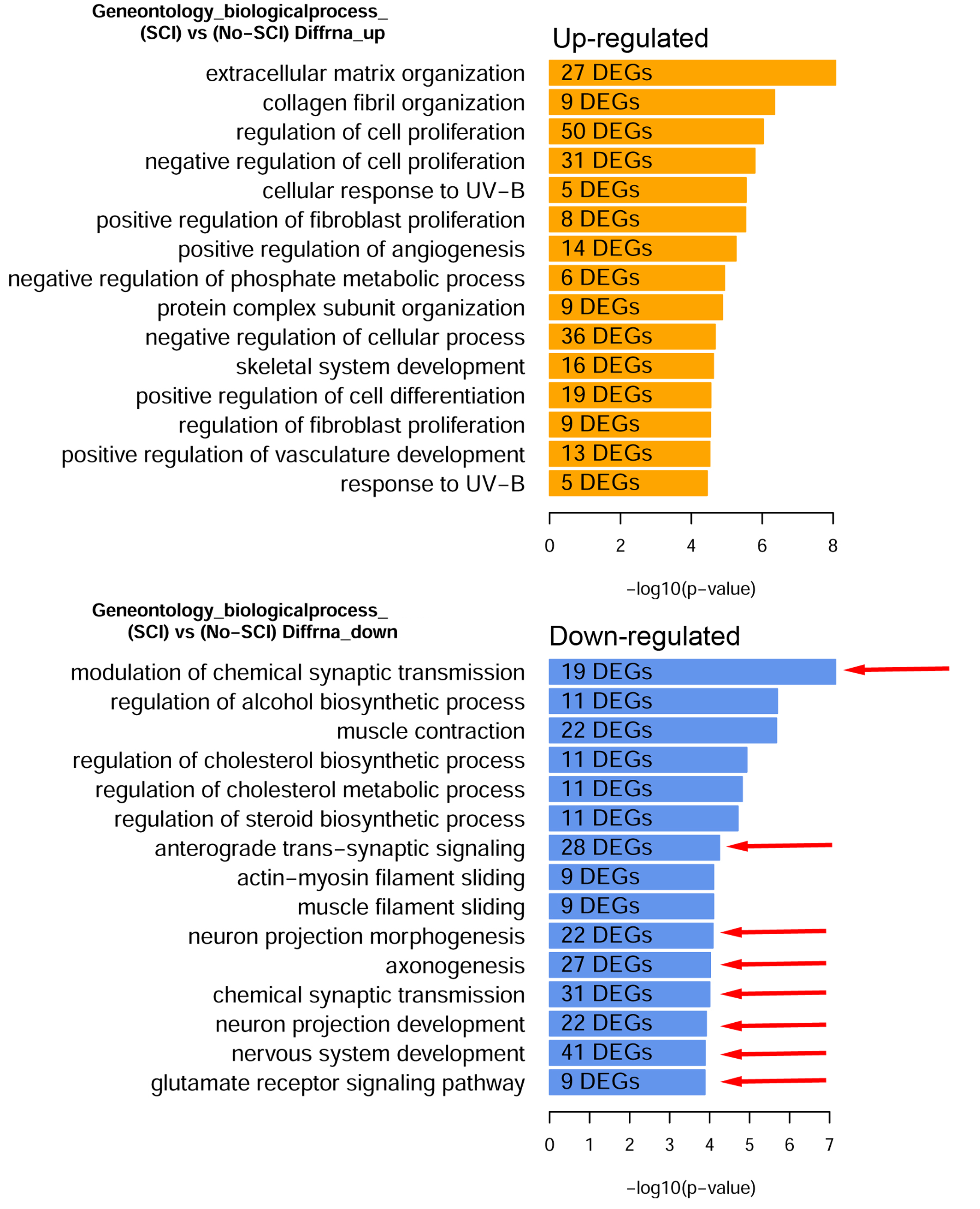

Supplement: SUPPLEMENTARY FIGURE 1 — Up- and down-regulated biological processes by GO analysis. From our RNA-seq we used GO analysis to determine biological processes that are being regulated. In the top figure in gold are pathways that are significantly up-regulated when comparing control to SCI. The lower figure in blue are pathways that are significantly downregulated. The red arrows are pointing to pathways known to be involved with neurons. [file Image_1.TIF]

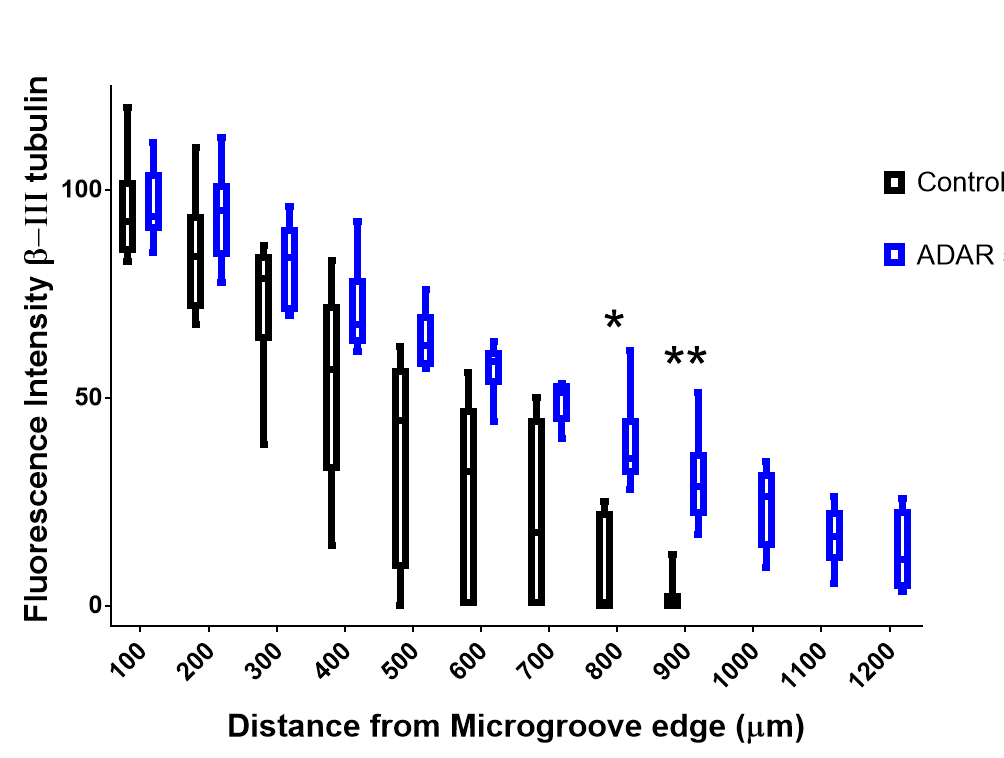

Supplement: SUPPLEMENTARY FIGURE 2 — siRNA against ADAR1 compared to scrambled controls promote longer axons in rat primary cortical neurons- Graph from Figure 6C is replotted to show the distribution of points. This graph is the average of four independent experiments with standard deviations. Statistics is t-test (*p < 0.05 and **p < 0.01) comparing Control to ADAR siRNA (blue) at a fixed distance in the microgrooves. At 800 and 900 microns, DAR siRNA (blue) were significantly longer compared to controls. [file Image_2.TIF]

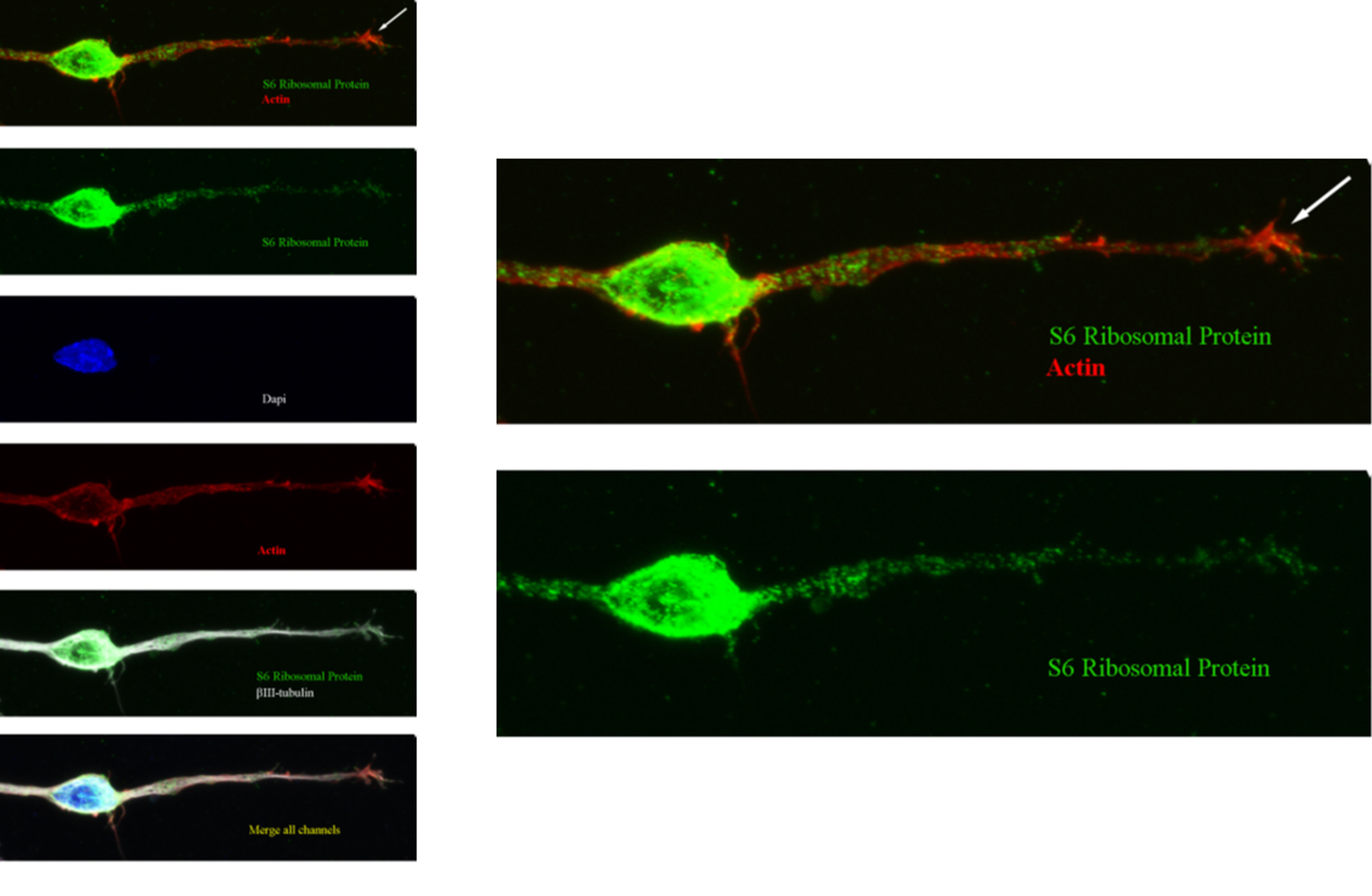

Supplement: SUPPLEMENTARY FIGURE 3 — Detection of ribosomes in the axons and growth cone tips of rat cortical neurons. We confirm expression of ribosomes in our cortical neurons by immunohistochemistry using an antibody against S6 Ribosomal Protein (green). We use Actin-Red to visualize the growth cones (white arrow). Dapi (blue) is staining the cell bodies and β-III tubulin immunohistochemistry to see the axons is in white. [file Image_3.TIF]

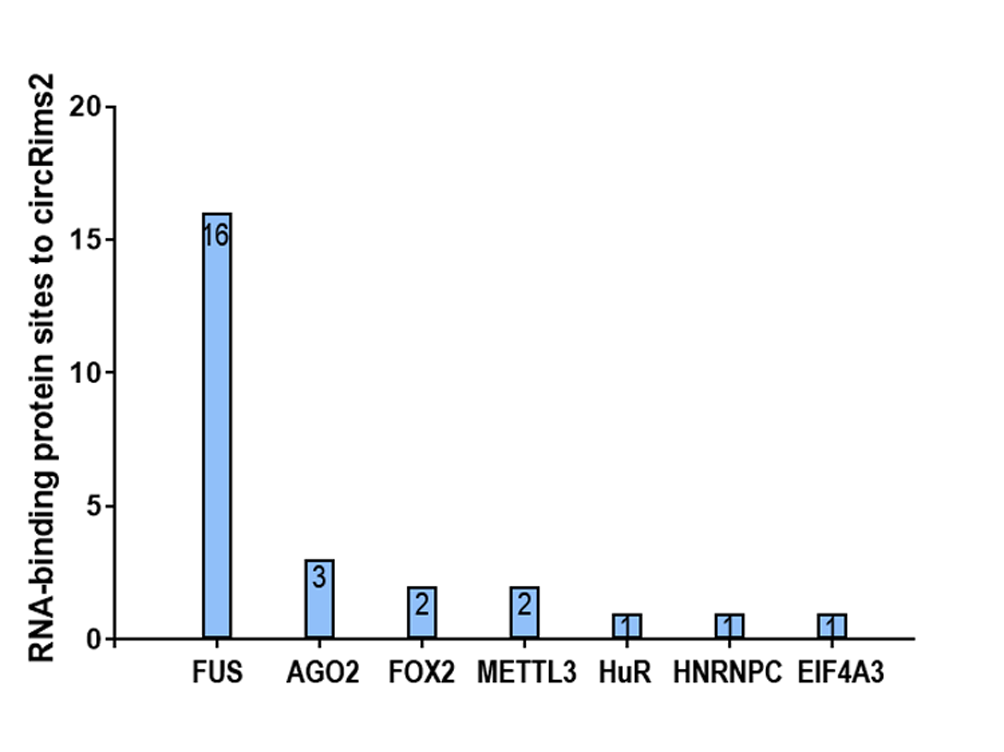

Supplement: SUPPLEMENTARY FIGURE 4 — Prediction of 7 RBPs binding to circRims2. Using Circular RNA Interactome we predicted 7 RBPs that could bind to circRims2. The number in each column represents the number of binding sites. Fused in Sarcoma (FUS) has 16 binding sites on circRims2. FUS has been suggested to be involved in neurite outgrowth and axonal transport (70–73). [file Image_4.TIF]
