# Supplementary material for: Spinal cord injury regulates circular RNA expression in axons
Source: Front Mol Neurosci. 2023 Aug 24;16:1183315. doi: 10.3389/fnmol.2023.1183315 (PMC10483835; doi:10.3389/fnmol.2023.1183315)
Supplement: Supplementary file 5 [file Table_1.DOCX]

**Supplementary Table 1 – Additional markers for spinal interneurons and motor neurons.** These are additional markers for spinal interneurons and motor neurons that have been reported. They are detected in low abundance. Intriguingly, ESRRG which was significantly down-regulated after injury is also a putative circRNA. Adjusted P value and fold change are given.
